# The Effect of Chinese Herbal Medicine Gualouxiebaibanxia Decoction for the Treatment of Angina Pectoris: A Systematic Review

**DOI:** 10.1155/2016/8565907

**Published:** 2016-09-29

**Authors:** Wei Liu, Xingjiang Xiong, Xiaochen Yang, Fuyong Chu, Hongxu Liu

**Affiliations:** ^1^Department of Cardiology, Beijing Hospital of Traditional Chinese Medicine, Capital Medical University, Beijing 100010, China; ^2^Department of Cardiology, Guang'anmen Hospital, China Academy of Chinese Medical Sciences, Beijing 100053, China

## Abstract

We systematically assess the current clinical evidence of Gualouxiebaibanxia (GLXBBX) decoction for the treatment of angina pectoris (AP). We included RCTs testing GLXBBX against conventional drugs and GLXBBX combined with conventional drugs versus conventional drugs. 19 RCTs involving 1730 patients were finally identified, and the methodological quality was evaluated as generally low. The results of the meta-analysis showed that GLXBBX alone had significant effect on improving angina symptoms (RR: 1.24, 95% CI 1.14 to 1.35; *P* < 0.00001), ECG (RR: 1.28 [1.13,1.44]; *P* < 0.0001), and HDL-C (MD: 0.56 [0.54,0.58]; *P* < 0.00001) compared with anti-arrhythmic drugs. A significant improvement in angina symptoms (RR: 1.17 [1.12,1.22]; *P* < 0.00001) and ECG (RR = 1.22; 95% CI = [1.14,1.30]; *P* < 0.00001) was observed for GLXBBX plus conventional drugs when compared with conventional drugs. Eight trials reported adverse events without serious adverse effects. GLXBBX appears to have beneficial effects on improvement of ECG and reduction of angina symptoms in participants with AP. However, the evidence remains weak due to the poor methodological quality of the included studies. More rigorous trials are needed to confirm the results.

## 1. Introduction

Coronary artery disease (CAD) is one of the main causes of morbidity and mortality worldwide [1–3]. CAD was the most common cause of death globally in 2013, resulting in 8.14 million deaths (16.8%) up from 5.74 million deaths (12%) in 1990 [4]. Coronary heart disease burden is projected to rise from around 47 million disability-adjusted life years (DALYs) globally in 1990 to 82 million DALYs in 2020 [5]. Nearly 58% of patients with coronary artery disease were suffering from chronic stable angina [6]. In every one million people in the general population of most European countries, it is estimated that 20,000 to 40,000 individuals suffer from angina pectoris (AP) [7, 8].

Angina significantly limits the ordinary activities of most of these patients and worsens their quality of life (QOL), in terms of not only physical activity/pain but also mental health [9, 10]. Current treatment strategies aim to reduce the risk of mortality and morbid events, reduce symptoms, and improve QOL [3, 11]. Despite multiple treatment options including pharmacotherapy (as organic nitrates, *β*-blockers, and calcium channel antagonists), revascularization, lifestyle changes, and aggressive management of modifiable coronary artery disease risk factors [11, 12], a high proportion of patients with stable angina remains symptomatic and widely used antianginal therapies have side effects, including headache, postural hypotension, and dizziness, and the continuous uptake of nitrates may lead to drug tolerance [13, 14]. Perhaps, there has been a growing interest in alternative therapies for AP.

Gualouxiebaibanxia (GLXBBX) decoction, which originated in the Eastern Han Dynasty (25–220), is a traditional Chinese medicinal herbal containing four commonly used herbs (*Trichosanthes kirilowii* Maxim.,* Allium macrostemon*, and white spirit). It has long been used to treat chest pain in clinical practice in China. Its mechanism of action could be related to promoting Qi to activate blood and removing blood stasis, activating Yang, and eliminating phlegm based on the theory of Traditional Chinese Medicine (TCM). Recent studies revealed that GLXBBX could dilate coronary arteries, antiplatelet aggregative activity, and antioxidant and hypolipidemic effect and ameliorates myocardial fibrosis [15–18]. Currently, there have been numbers of research works indicating that GLXBBX is effective to AP, whereas the data supporting the validity is not enough. This systematic review aims to assess the current clinical evidence for the efficacy of GLXBBX for the treatment of AP by integrating different outcome measures from randomized controlled trials (RCTs).

## 2. Material and Methods

### 2.1. Information Sources and Search Strategies

A systematic search was conducted in the following 7 online electronic databases from their inception until January 20, 2016: Cochrane Central Register of Controlled Trials (CENTRAL, 1996–2016), PubMed (1959–2016), EMBASE (1980–2016), Chinese Scientific Journal Database (VIP, 1989–2016), Chinese Biomedical Literature Database (1978–2016), Wanfang database (WMOD, 1985–2016), and Chinese National Knowledge Infrastructure (CNKI, 1979–2016). In addition, as GLXBBX is mainly prescribed in China, we also conducted a literature search of the website of the Chinese Clinical Trial Registry (available at http://www.chictr.org.cn/index.aspx); the reference lists of retrieved articles were also reviewed. No restriction on publication status or language was imposed. Search terms used were (“angina pectoris” OR “coronary artery disease”) AND (“herb” OR “Gualouxiebaibanxia Decoction” OR “GuaLouXieBaiBanXia” OR “Gualouxiebaibanxia Tang”) AND (“randomized controlled trial” OR “controlled clinical trial” OR “random” OR “randomly” OR “randomized” OR “control”).

### 2.2. Eligibility Criteria

Studies that met the following criteria were included: (1) GLXBBX combined with conventional drugs versus conventional drugs; (2) GLXBBX versus conventional drugs; and (3) duration of treatment being at least 2 weeks. The participants who were suffering from and had been diagnosed with AP should be included regardless of the severity. The primary outcome measures in RCTs were mortality due to ischemic heart disease or incidence of heart events; the secondary outcome measure was reduction of angina symptoms (RAS) and ECG improvements. Other outcomes like frequency of angina attack (FAA), blood lipid, follow-up, and adverse events were also measured. Duplicated publications reporting the same groups of participants were excluded. The clinical standards of AP are by “the International Society and Federation of Cardiology/World Health Organization (ISFC/WHO) [38]” or “ACC/AHA 2002 guideline update for the management of patients with chronic stable angina task force on practice guidelines (2002 ACCF/AHA) [39].” Effective symptomatic improvements should achieve at least 50% (basic) or 80% (significant) reduction of angina symptoms (RAS) [38]. Effective ECG improvements should achieve at least 0.05 mV lowering at ST segment in ECG (basic) or nearly normal (significant) ECG during an exercise test [38].

### 2.3. Study Selection and Data Collection Process

Two reviewers (Wei Liu and Xingjiang Xiong) independently screened the titles, abstracts, and key words of each searched article according to the eligibility criteria. One author (Xiaochen Yang) extracted data from the included RCTs and then put them into Microsoft Excel. Another 2 authors (Wei Liu and Fuyong Chu) examined the accuracy of extracted data. The data extraction form comprised the authors, title, publication year, sample size, age, sex distribution, diagnosis standard, study design, interventions in the treatment and control groups, composition of GLXBBX or modified GLXBBX, trial duration, outcome measures, and adverse effects. If there were discrepancies in the process of selection, whether to include or exclude a study was resolved by a third author (Hongxu Liu).

### 2.4. Quality Assessment

The methodological quality of trials was assessed independently by two authors (Xiaochen Yang and Xingjiang Xiong) using criteria from the Cochrane Handbook for Systematic Review of Interventions [40]. The qualities of included RCTs were assessed by six specific domains, including random sequence generation, allocation concealment, blinding of participants and personnel, blinding of outcome data, incomplete outcome data, and selective reporting. We judged each item from three levels (“yes” for a low risk of bias, “no” for a high risk of bias, and “unclear” otherwise). Then the methodological quality of the trials was ranked into three levels: low risk of bias (all items with low risk of bias), high risk of bias (at least one item with high risk of bias), or unclear risk of bias (at least one item with an unclear domain).

### 2.5. Statistical Analysis

Meta-analyses of RCTs were performed by using RevMan 5.1 software (The Nordic Cochrane Centre, The Cochrane Collaboration, Copenhagen, 2011). Dichotomous data were expressed as relative risk (RR) or continuous outcomes as weighted mean difference (WMD), both with 95% confidence intervals (CI). Subgroups analysis was conducted among different types of comparisons (including GLXBBX versus conventional drugs and GLXBBX plus conventional drugs versus conventional drugs). Heterogeneity between trials was recognized as significant when *I* ^2^ > 50% or *P* < 0.1. The fixed effects model was used to analyze data with low heterogeneity (heterogeneity test, *P* > 0.10), whereas the random effects model was applied if heterogeneity was significant (heterogeneity test, *P* < 0.10). Publication bias was assessed by funnel plot analysis if the group included more than 10 trials [40].

## 3. Results

### 3.1. Study Identification

**Figure 1:**
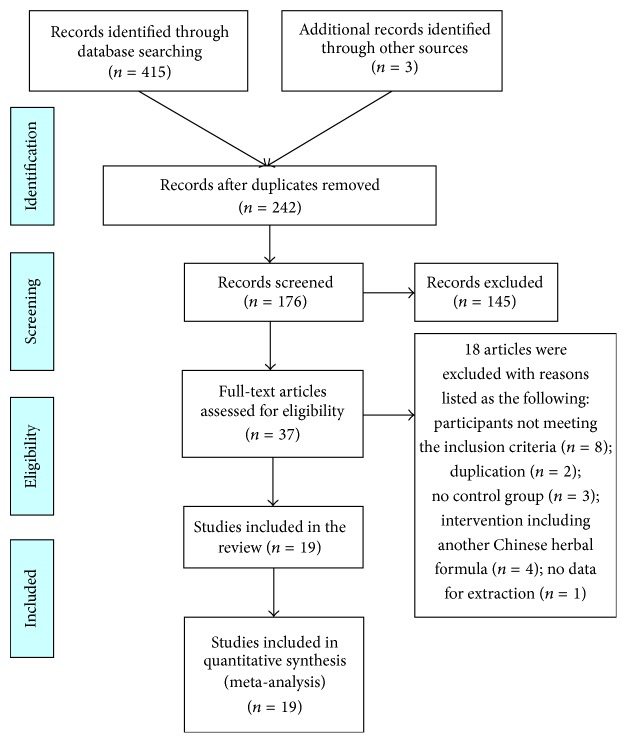
Flow diagram of study selection and identification.

An initial screening yielded 418 potentially relevant citations in accordance with the search strategy. A total of 176 articles were screened after 242 duplicates of the same articles included in different databases were removed. According to the inclusion criteria, 145 articles were excluded on the basis of the titles and abstracts. These studies were primarily excluded because they were not RCTs. A total of 37 full-text articles were retrieved for further assessment, of which 18 were excluded for the following reasons: participants not meeting the inclusion criteria (*n* = 8); duplication (*n* = 2); no control group (*n* = 3); intervention including another Chinese herbal formula (*n* = 4); and no data for extraction (*n* = 1). In the end, 19 RCTs were included, and all trials had been conducted and published in China. A flow chart depicted the search process and study selection (as shown in Figure 1).

### 3.2. Study Characteristics

The 19 RCTs [19–37], which involved a total number of 1730 patients with angina pectoris, ranging from 60 to 210, were published between 2001 and 2015. The age of the angina pectoris patients ranged from 35 to 90 years. The duration of treatment varied from 2 weeks to 8 weeks. The dosage of GLXBBX was one dose twice a day. Three diagnostic criteria of AP were specified: six trials [19, 25, 27, 29, 32, 37] used the Guidelines of Clinical Research of New Drugs of Traditional Chinese Medicine (GCRNDTCM); ten trials [20–22, 24, 28, 30, 31, 33, 34, 36] used the International Society and Federation of Cardiology/World Health Organization-1979 (1979 ISFC/WHO); one trial [35] used “ACCF/AHA Guideline for the Diagnosis and Management of Patients with Unstable Ischemic Heart Disease-2002 (2002 ACCF/AHA). There were two comparisons: 6 trials [19–24] compared GLXBBX and conventional drugs alone and 13 trials [25–37] compared the combination of GLXBBX and conventional drugs with conventional drugs. Reductions in angina symptoms and improvement in ECG were the most commonly measured outcomes in the included studies. The other outcomes included changes of blood lipid and frequency of angina attack. Eight trials reported adverse events [19, 24, 25, 27, 33, 35–37]. The descriptive information of the included trials and subjects in this review was summarized in Table 1 and GLXBBX's dosages and compositions are listed in Table 2.

### 3.3. Study Quality

**Figure 2:**
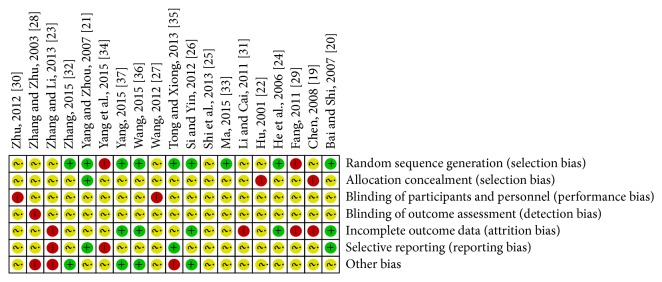
Risk of bias summary: reviewing authors' judgments about each risk of bias item for each included study.

**Figure 3:**
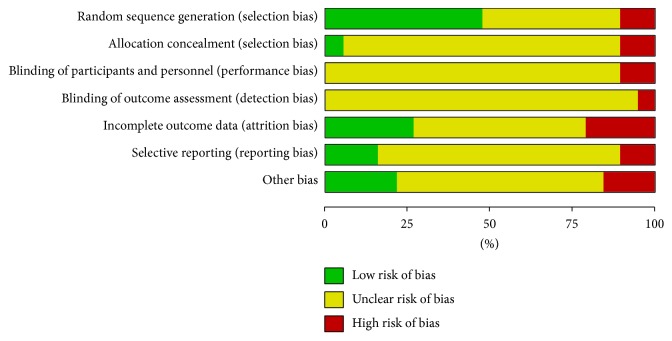
Risk of bias graph: reviewing authors' judgments about each risk of bias item presented as percentages across all included studies.

Among trials, only 9 studies [21, 24, 26, 32, 33, 35–37] stated the method of the sequence generation with random number table and drawing [20], while none of the 19 studies reported details for sample size calculations and none was double-blind, placebo controlled study. Additionally, none mentioned allocation concealment or blinding methods. None reported information on follow-up and two trials [33, 35] had reported dropout without explaining their reasons. Among all RCTs, the characteristics of participants in each study arm were similar at baseline (age, race, sex, and disease course). Selective reporting could not be evaluated as no preregistered protocols could be obtained from the primary authors. The details of the risk of bias of each trial are presented in Figures 2 and 3.

### 3.4. Effects of the Interventions

There was no report of mortality as the primary outcome measures (e.g., AMI, severity arrhythmia, heart failure, and revascularization). We analyzed the outcomes, RAS (19 trials), ECG (13 trials), and blood lipid (HDL-C, LDL-C) level (4 trials), and used subgroup analysis with consideration of clinical heterogeneity across the studies. 19 studies were divided into two groups: one evaluated the effects of GLXBBX versus conventional drugs; the other compared GLXBBX plus conventional drugs versus conventional drugs alone.

#### 3.4.1. Reduction of Angina Symptoms (RAS)

**Figure 4:**
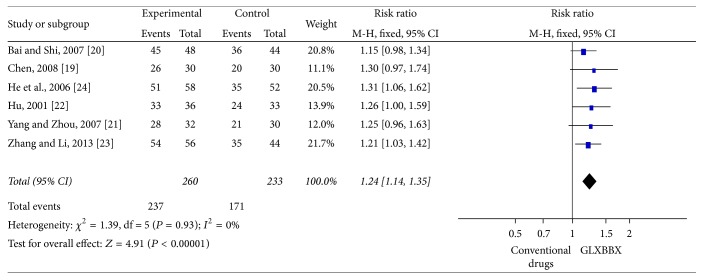
Analysis of RAS for AP. Forest plot of comparison: GLXBBX versus conventional drugs.

**Figure 5:**
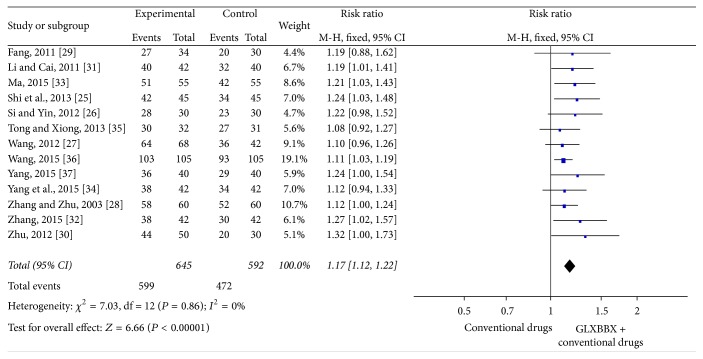
Analysis of RAS for AP. Forest plot of comparison: GLXBBX combined with conventional drugs versus conventional drugs.

All the 19 trials [19–37] reported the RAS for AP. Six trials [19–24] compared GLXBBX with conventional drugs. Homogeneity in the results is shown (*χ* ^2^ = 1.39, df = 5 (*P* = 0.93), *I* ^2^ = 0%). Thus, we did a quantitative data synthesis (meta-analysis) by fixed effects model. The meta-analysis showed that there is significant beneficial effect on the GLXBBX group compared to conventional drugs using alone (RR: 1.24, 95% CI 1.14 to 1.35; *P* < 0.00001) (Figure 4). The improvement of RAS was reported in 13 RCTs [25–37] involving 1237 participants and results favored GLXBBX combined with conventional drugs (RR: 1.17 [1.12,1.22]; *P* < 0.00001) without significant heterogeneity (*χ* ^2^ = 7.03, df = 12, *I* ^2^ = 0%) (Figure 5).

#### 3.4.2. ECG Improvement

**Figure 6:**
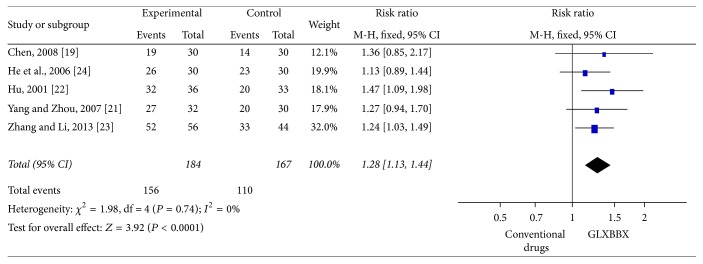
Analysis of ECG improvement for AP. Forest plot of comparison: GLXBBX versus conventional drugs.

**Figure 7:**
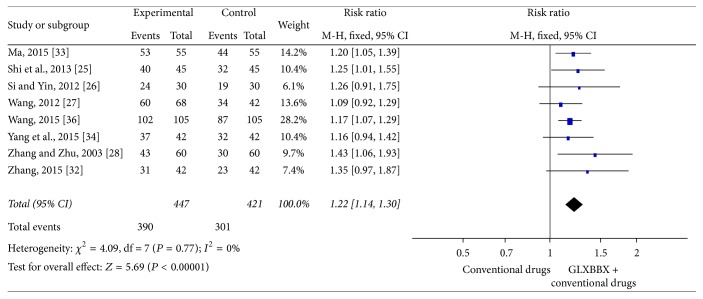
Analysis of ECG improvement for AP. Forest plot of comparison: GLXBBX combined with conventional drugs versus conventional drugs.

13 RCTs evaluated the effect of ECG improvement [19, 21–28, 32–34, 36]. ECG was significantly improved in the GLXBBX group when compared with conventional drugs (RR: 1.28 [1.13, 1.44]; *P* < 0.0001, Figure 6), with no significant heterogeneity (*χ* ^2^ = 1.98, *P* = 0.74; *I* ^2^ = 0%). After analyzing 8 RCTs [25–28, 32–34, 36] involving 868 participants, the result also indicated favoring GLXBBX combined with conventional drugs group (RR = 1.22; 95% CI = [1.14,1.30]; *P* < 0.00001) in the improvement of ECG and with significant homogeneity (*χ* ^2^ = 4.09; *I* ^2^ = 0%). The effect estimates were shown in Figure 7.

#### 3.4.3. Other Outcomes (Frequency of Angina Attack and Blood Lipid)

**Figure 8:**
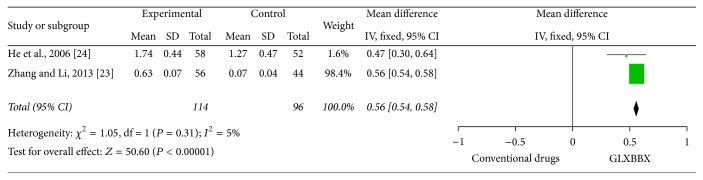
Forest plot of comparison: GLXBBX versus conventional drugs, outcome: HDL-C.

Compared with conventional medicine, one trial [27] indicated that frequency of angina attack decreased significantly (*P* < 0.05) in GLXBBX plus conventional drugs group. Two trials [23, 24] reported the improvement of HDL-C of 210 patients after 4 weeks of treatment with GLXBBX alone. The statistical data show that GLXBBX was better than conventional drugs alone (MD: 0.56 [0.54,0.58]) with significant homogeneity (*χ* ^2^ = 1.05; *I* ^2^ = 5%). The effect estimates were shown in Figure 8.

Compared with conventional drugs, 2 RCTs [23, 24] reported the decline levels of LDL-C after treatment favored GLXBBX. LDL-C was significantly decreased in GLXBBX but with significant heterogeneity (*P* = 0.001, *I* ^2^ = 91%). Thus, we did not adopt a meta-analysis. Zhang and Li [23] indicated that duration of angina attack which decreased from 3.52 ± 1.31 mmol/L to 1.53 ± 0.75 mmol/L after treatment favored GLXBBX. One trial [24] showed that after treatment the level of LDL-C decreased significantly (*P* < 0.05) in GLXBBX group compared to conventional drugs.

Compared with conventional medicine, two individual trials [29, 35] reporting LDL-C after treatment favored GLXBBX plus conventional medicine. No homogeneity in the results is shown (*P* < 0.00001, *I* ^2^ = 96%). Thus, we did not adopt a meta-analysis. Fang [29] indicated that LDL-C which decreased from 3.74 ± 0.85 mmol/L to 1.92 ± 0.67 mmol/L after treatment favored GLXBBX plus conventional medicine. Another trial [35] also indicated after treatment favored GLXBBX plus conventional medicine: LDL-C decreased from 4.65 ± 1.24 mmol/L to 2.56 ± 0.72 mmol/L.

### 3.5. Subgroup Analysis

Three subgroups were analyzed based on methodological variables of different AP subtypes; 13 studies provided the data necessary to perform our evaluation. Of these RCTs, 2 RCTs [26, 35] involved participants with UAP, 3 RCTs [28, 29, 36] involved participants with SAP, and the other 8 RCTs [25, 27, 30–34, 37] involved participants with SAP or UAP. Overall, (1) for patients with diagnosis of either SAP or UAP, 91.93% reported RAS improvement in the experimental group compared with 76.49% in the control group (therapeutic gain = 15.44% with an NNT = 6.48) (Table 3), and ECG improvement was 87.5% versus 71.20% (therapeutic gain = 16.30% with an NNT = 6.13) (Table 4). (2) For patients with SAP, 94.47% reported RAS improvement after the treatment with GLXBBX plus conventional drugs compared with 84.62% after the treatment with conventional drugs only (therapeutic gain = 9.85% with a number needed to treat NNT = 10.15) (Table 5) and 87.88% compared with 70.91% in ECG improvement (therapeutic gain = 16.98% with an NNT = 5.89) (Table 6). (3) For patients with UAP, 93.55% reported RAS improvement in the experimental group compared with 81.97% in the control group (therapeutic gain = 11.59% with an NNT = 8.63) (Table 7), and ECG improvement was 80.00% versus 63.33% (therapeutic gain = 16.67% with an NNT = 6.00) (Table 8).

### 3.6. Adverse Effect

A total of 8 trials [19, 24, 25, 27, 33, 35–37] mentioned the occurrence of adverse effects. The four studies [19, 25, 36, 37] reported that 4 patients with nausea (4/145, 2.76%) were identified in the GLXBBX combined with conventional drugs group, whereas 8 patients with gastrointestinal reaction (8/175, 4.57%) and 12 patients with headache (12/115, 10.43%) were observed in the conventional drugs group. And the remaining 4 trials [24, 27, 33, 35] reported that no adverse effects occurred. No severe adverse events were reported.

### 3.7. Publication Bias

**Figure 9:**
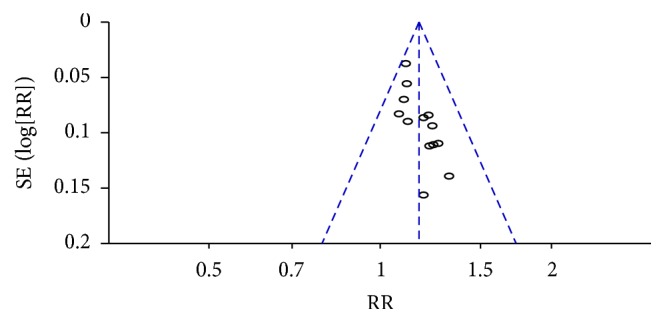
Funnel plot of comparison: GLXBBX combined with conventional drugs versus conventional drugs for the outcome of RAS.

The forest plot of comparison of GLXBBX combined with conventional drugs and conventional drugs for the outcome of RAS was shown in Figure 9.

## 4. Discussion

### 4.1. Summary of Evidences

Angina pectoris (AP) is a highly prevalent condition in persons with known coronary artery disease (CAD), and the burden of cardiovascular (CV) disease (CVD) remains high, with more than 2200 Americans dying of CVD every day [41, 42]. The aim of management is to abolish or minimise symptoms and to improve quality of life and long-term morbidity and mortality [43]. Most patients improved ECG and symptoms by conventional treatment of western medicine. However, the current status of treatment is unsatisfactory [44]. Therefore, it is very important to seek for more safe and effective prevention and treatment. Recently, with the growing and sustained interest in the benefits of traditional Chinese herbs and integrative medicine, GLXBBX is widely used to treat AP in clinical practice for a long time in China. Meanwhile, there have been a large number of fundamental research and clinical trials of GLXBBX on AP and RCTs; however, no high level of evidence such as systematic review or meta-analysis was provided for further recommendation. To the best of our knowledge, this is the first SR of GLXBBX D in English.

Nineteen claimed RCTs, with a total of 1730 patients with AP, met the inclusion criteria and were included in this review. The results suggested that RAS and ECG were significantly improved in patients receiving GLXBBX plus conventional drugs therapy or GLXBBX alone. The combination therapy of GLXBBX and conventional drugs could also reduce the frequency of angina attack. In recent years, there are many studies proving the role of lipid profile fractions for the development of coronary artery disease (CAD) [45, 46]. Previous studies demonstrated that endothelial dysfunction and increased oxidative stress are associated with the dysfunction and dysregulation of individual lipids [47–50]. Some clinical trials and basic researches showed the arterial stiffness correlated positively with specific lipid and oxidative stress and the triacylglycerol (TAG), very-low-density lipoprotein (VLDL), and total cholesterol/high-density lipoprotein (TC/HDL) were significantly affecting the severity of myocardial damage in the patients of UAP [51, 52]. Therefore, lowering blood lipid level is one of the important measures to protect the CVD. In this review, GLXBBX can effectively decrease blood LDL-C combined with conventional drugs. Moreover, GLXBBX was found to be effective in terms of improving blood HDL-C level which benefits patients with CVD and decreases blood LDL-C level, when compared with conventional drugs. This result is encouraging which indicates new optional treatment for AP, but the methodological quality of the trials was evaluated generally as low, and the conclusion needs to be confirmed by further study.

### 4.2. Limitations

Firstly, the following problems reported contribute to the limited methodological quality of most included trials. Although all studies claimed randomization, only 9 studies [21, 24, 26, 32, 33, 35–37] stated the method of the sequence generation with random number table and drawing [20], and the other 10 trials just mentioned “randomly allocating” with no detailed information. In addition, all the trials did not describe allocation concealment in detail and blinding of participants and personnel and blinding of outcome assessment were unclear, which lead to inability to judge whether the study was conducted properly. No multicenter, large-scale RCTs were identified and none of trials had a pretrial estimation of sample size, which indicated the lack of statistical power to ensure appropriate estimation of the therapeutic effect. No trial reported information on follow-up; AP can typically recur across the life span and so long-term follow-up is required for accurate analysis. Only two trials [33, 35] had reported dropout with the detailed reasons, which might have led to attrition bias and other biases.

Secondly, heterogeneity is another critical issue that should be considered. The 4 independent trials [23, 24, 29, 35] using GLXBBX alone or combined with conventional drugs compared with conventional drugs did show significant heterogeneity in the results of blood LDL-C level. The significant clinical heterogeneity reflected in variations in methodological quality, participants, interventions, and conventional drugs might weaken the reliability of the data [53]. Four trials specified 3 diagnostic criteria with different types of angina (SAP, UAP) and the durations of treatment were various (ranged from 3 to 8 weeks). In addition, the methodological quality of the trials [23, 24, 29, 35] included was different; 2 RCTs [24, 35] were scored as having superior quality, which mentioned random sequence generation. As a result, the limited number of included trials and different interventions in the GLXBBX and conventional drugs groups restricted us from conducting meaningful subgroup analyses.

Thirdly, none of the included trials reported severe adverse events possibly related to GLXBBX, and the adverse effects included 4 cases with nausea, 2.76% (4/145), 8 cases with gastrointestinal reaction, 4.57% (8/175), and 12 cases with headache, 10.43% (12/115), and these side effects may be related to the adverse effect of conventional drugs. Safety is a serious concern that should be recorded in detail. Thus, definitive conclusions about the safety of GLXBBX still cannot be drawn since 4 of 19 trials did report information on safety.

Fourthly, Figure 9 was asymmetrical, which indicated that publication bias might influence the results of our analysis. Moreover, while GLXBBX is a widely used therapy for AP in China, positive results were reported in most of the included studies and some negative results could not be reported, so a certain degree of potential selective bias might exist in this conclusion.

Last but not least, apart from the limitations on the mediocre methodological quality of included studies, the inadequate reporting of mortality or the incidence of complications was another important limitation of this systematic review. Moreover, outcome measures of AP including RAS and ECG improvement and the blood lipid level and frequency of angina attack were limited by relatively small sample size. As a result, evaluating the efficacy of GLXBBX on AP requires more strictly designed large-scale randomized clinical trials.

## 5. Conclusion

From this systematic review, we find that patients receiving GLXBBX adjunct therapy alone or combined with conventional drugs could significantly improve RAS, ECG, the blood lipid level, and frequency of angina attack in patient with AP. But the previous results should be read with caution owing to the poor methodological quality and some possible biases. Thereby, in order to explore the efficacy and safety of GLXBBX treating AP, well-designed, complete efficacy indicator, larger scaled, and multicenter randomized clinical trials with long-term follow-up are warranted for stronger evidence in the future.

## Figures and Tables

**Table 1:** Characteristics and methodological quality of included studies.

Study ID	Sample (M/F)	Age (yrs)	Type of angina	Diagnosis standard	Intervention	Control	Course (week)	Outcome measure
Chen, 2008 [19]	60 T: 20/10; C: 18/12	T: 48–70 (52 ± 10) C: 50–70 (55 ± 10)	T: SAP: 20, UAP: 10 C: SAP 16, UAP: 14	GCRNDTCM	Modified GLXBBX decoction (1 dose/day)	Conventional drugs	4	RAS; ECG; adverse event

Bai and Shi, 2007 [20]	92 T: 48; C: 44	43–75	AP	1979 ISFC/WHO	Modified GLXBBX decoction (1 dose/day)	Conventional drugs	4	RAS

Yang and Zhou, 2007 [21]	62 T: 18/14; C: 17/13	T: 48–75 C: 46–77	AP	1979 ISFC/WHO	Modified GLXBBX decoction (1 dose/day)	Conventional drugs	4	RAS; ECG

Hu, 2001 [22]	69 T: 18/14; C: 17/13	T: 40–74 C: 41–73	AP	1979 ISFC/WHO	Modified GLXBBX decoction (1 dose/day)	Conventional drugs	4	RAS; ECG

Zhang and Li, 2013 [23]	100 T: 31/25; C: 27/17	T: 35–72 (56.5 ± 5.3) C: 50–70 (58.2 ± 6.2)	SAP: 71; UAP: 29	Unclear	Modified GLXBBX decoction (1 dose/day)	Conventional drugs	4	RAS; ECG; BL

He et al., 2006 [24]	110 T: 38/20; C: 31/21	T: 47–76 (59.25 ± 6.07) C: 45–78 (54.76 ± 3.83)	AP	1979 ISFC/WHO	Modified GLXBBX decoction (1 dose/day)	Conventional drugs	4	RAS; ECG; BL; adverse event

Shi et al., 2013 [25]	90 T: 23/22; C: 22/23	T: 40–68 (53.75) C: 41–69 (54.22)	AP	GCRNDTCM	Modified GLXBBX decoction (1 dose/day) + conventional drugs	Conventional drugs	4	RAS; ECG; adverse event

Si and Yin, 2012 [26]	60 T: 23/22; C: 22/23	T: 40–68 (53.75) C: 41–69 (54.22)	UAP	Unclear	Modified GLXBBX decoction (1 dose/day) + conventional drugs	Conventional drugs	2	RAS; ECG

Wang, 2012 [27]	110 T: 45/23; C: 26/16	T: 39–75 (62.6) C: 41–73 (59.8)	AP	GCRNDTCM	Modified GLXBBX decoction (1 dose/day) + conventional drugs	Conventional drugs	2	RAS; FAA; adverse event

Zhang and Zhu, 2003 [28]	120 T: 38/22; C: 40/20	T: 40–65 C: 41–63	SAP	1979 ISFC/WHO	Modified GLXBBX decoction (1 dose/day) + conventional drugs	Conventional drugs	4	RAS; ECG

Fang, 2011 [29]	64 T: 18/16; C: 15/15	T: 46–86 C: 45–90	SAP	GCRNDTCM	Modified GLXBBX decoction (1 dose/day) + conventional drugs	Conventional drugs	8	RAS; BL

Zhu, 2012 [30]	80 T: 28/22; C: 18/12	T: 40–82 C: 39–95	AP	1979 ISFC/WHO	Modified GLXBBX decoction (1 dose/day) + conventional drugs	Conventional drugs	8	RAS

Li and Cai, 2011 [31]	82 T: 27/15; C: 26/14	T: 48–76 C: 52–74	AP	1979 ISFC/WHO	Modified GLXBBX decoction (1 dose/day) + conventional drugs	Conventional drugs	4	RAS

Zhang, 2015 [32]	84 T: 25/17; C: 23/19	T: 62–86 (71.53 ± 5.26) C: 61–88 (70.64 ± 4.23)	AP	GCRNDTCM	Modified GLXBBX decoction (1 dose/day) + conventional drugs	Conventional drugs	2	RAS; ECG

Ma, 2015 [33]	110 T: 29/26; C: 27/28	T: 40–81 (53.73 ± 5.6) C: 40–80 (52.4 ± 5.3)	T: SAP: 14, UAP: 41 C: SAP 16, UAP: 39	1979 ISFC/WHO	Modified GLXBBX decoction (1 dose/day) + conventional drugs	Conventional drugs	8	RAS; ECG; adverse event

Yang et al., 2015 [34]	84 T: 26/16; C: 24/18	T: 55.9 ± 5.7 C: 56.7 ± 5.8	SAP: 30, UAP: 54	1979 ISFC/WHO	Modified GLXBBX decoction (1 dose/day) + conventional drugs	Conventional drugs	2	RAS; ECG

Tong and Xiong, 2013 [35]	63 T: 20/12; C: 18/13	T: 48–74 (61.77 ± 8.34) C: 45–75 (63.29 ± 8.64)	UAP	2002 ACCF/AHA	Modified GLXBBX decoction (1 dose/day) + conventional drugs	Conventional drugs	3	RAS; BL; adverse event

Wang, 2015 [36]	210 T: 66/39; C: 63/52	T: 45–70 (59.25) C: 45–70 (58.55)	SAP	1979 ISFC/WHO	Modified GLXBBX decoction (1 dose/day) + conventional drugs	Conventional drugs	4	RAS; ECG; adverse event

Yang, 2015 [37]	80 T: 22/18; C: 24/16	T: 39–78 (58.3 ± 5.7) C: 41–82 (56.8 ± 6.2)	AP	GCRNDTCM	Modified GLXBBX decoction (1 dose/day) + conventional drugs	Conventional drugs	8	RAS; adverse event

T, intervention group; C, control group; SAP: stable angina pectoris; UAP: unstable angina pectoris; RAS: reduction of angina symptoms; FAA: frequency of angina attack; GCRNDTCM: Guidelines of Clinical Research of New Drugs of Traditional Chinese Medicine; BL: blood lipid.

**Table 2:** Compositions of GLXBBX decoction in the included trials.

Study ID	Formula	Composition of formula
Chen, 2008 [19]	Modified GLXBBX decoction	Snake Gourd Fruit (Gualou, *Trichosanthes kirilowii* Maxim.) 10 g, *Allium macrostemon* Bunge (Xiebai, *Allium macrostemon*) 10 g, *Pinellia ternata* (Banxia, Rhizoma Pinelliae) 10 g, Fruit of Trifoliate Orange (Zhiqiao, Fructus Aurantii) 10 g, Tangerine Peel (Chenpi, Pericarpium Citri Reticulatae) 10 g, *Salvia *Root (Danshen, Radix Salviae Miltiorrhizae) 10 g, Notoginseng Root (Sanqifen, *Panax notoginseng* powder) 6 g, White Atractylodes Rhizome (Baizhu, Rhizoma Atractylodis Macrocephalae) 20 g, Chinese Angelica Root (Danggui, Radix Angelicae Sinensis) 10 g, Liquorice Root (Gancao, Radix Glycyrrhizae) 6 g

Bai and Shi, 2007 [20]	Modified GLXBBX decoction	Snake Gourd Fruit (Gualou, *Trichosanthes kirilowii* Maxim.) 15 g, *Allium macrostemon* Bunge (Xiebai, *Allium macrostemon*) 12 g, *Salvia *Root (Danshen, Radix Salviae Miltiorrhizae) 30 g, Heterophylly Falsestarwort Root (Taizishen, Radix Pseudostellariae) 30 g, *Pinellia ternata* (Banxia, Rhizoma Pinelliae) 12 g, Szechuan Lovage Root (Chuanxiong, Rhizoma Ligustici Chuanxiong) 12 g, *Trogopterus* Dung (Wulingzhi, Faeces Trogopterori) 12 g, Chinese Angelica Root (Danggui, Radix Angelicae Sinensis) 12 g, Sclerotium of Tuckahoe (Fuling, Scierotium Poriae Cocos) 12 g, Cassia Twig (Guizhi, Ramulus Cinnamomi) 6 g, White Sandalwood (Tanxiang, Sandalwood) 6 g, Amomum Fruit (Sharen, *Amomum villosum*) 6 g

Yang and Zhou, 2007 [21]	Modified GLXBBX decoction	Snake Gourd Fruit (Gualou, *Trichosanthes kirilowii* Maxim.) 30 g, *Allium macrostemon* Bunge (Xiebai, *Allium macrostemon*) 15 g, *Pinellia ternata* (Banxia, Rhizoma Pinelliae) 10 g, Pueraria (Gegen, Radix Puerariae) 20 g, Ginseng (Renshen, Panax ginseng) 12 g, *Salvia *Root (Danshen, Radix Salviae Miltiorrhizae) 20 g, Chinese Motherwort (Yimucao, Herba Leonuri Heterophylli) 30 g, Chinese Thorowax Root (Chaihu, Radix Bupleuri) 10 g, Fruit of Trifoliate Orange (Zhiqiao, Fructus Aurantii) 10 g, Turmeric Tuber (Yujin, Tuber Curcumae) 10 g, *Corydalis* Rhizome (Yanhusuo, *Corydalis* Rhizome) 15 g, Root of Common Peony (Chishao, Radix Paeoniae Rubra) 15 g, White Peony Root (Baishao, Radix Albus Paeoniae Lactiflorae) 15 g

Hu, 2001 [22]	Modified GLXBBX decoction	Snake Gourd Fruit (Gualou, *Trichosanthes kirilowii *Maxim.) 15 g, *Allium macrostemon* Bunge (Xiebai, *Allium macrostemon*) 15 g, *Pinellia ternata* (Banxia, Rhizoma Pinelliae) 10 g, *Salvia *Root (Danshen, Radix Salviae Miltiorrhizae) 10 g, Notoginseng Root (Sanqi, Notoginseng Radix) 6 g, Turmeric Tuber (Yujin, Tuber Curcumae) 10 g, Peach Seed (Taoren, Peach Keruel) 10 g, Safflower (Honghua, *Carthamus tinctorius*) 10 g, Leech (Shuizhi, *Hirudo nipponia* Whitman) 6 g, *Codonopsis* Root (Dangshen, Radix Codonopsitis Pilosulae) 12 g, *Acorus gramineus* (Shichangpu, Rhizoma Acori Tatarinowii) 5 g, Liquorice Root (Gancao, Radix Glycyrrhizae) 6 g

Zhang and Li, 2013 [23]	Modified GLXBBX decoction	Snake Gourd Fruit (Gualou, *Trichosanthes kirilowii *Maxim.) 25 g, *Allium macrostemon* Bunge (Xiebai, *Allium macrostemon*) 15 g, *Pinellia ternata* (Banxia, Rhizoma Pinelliae) 10 g, Ginseng (Renshen, Panax ginseng) 10 g, Pueraria (Gegen, Radix Puerariae) 25 g, *Salvia *Root (Danshen, Radix Salviae Miltiorrhizae) 15 g, Cassia Twig (Guizhi, Ramulus Cinnamomi) 10 g, Chinese Thorowax Root (Chaihu, Radix Bupleuri) 10 g, Turmeric Tuber (Yujin, Tuber Curcumae) 10 g, *Corydalis* Rhizome (Yanhusuo, *Corydalis* Rhizome) 10 g, White Peony Root (Baishao, Radix Albus Paeoniae Lactiflorae) 20 g

He et al., 2006 [24]	Modified GLXBBX decoction	Snake Gourd Fruit (Gualou, *Trichosanthes kirilowii *Maxim.) 25 g, *Allium macrostemon* Bunge (Xiebai, *Allium macrostemon*) 15 g, *Pinellia ternata* (Banxia, Rhizoma Pinelliae) 10 g, Ginseng (Renshen, Panax ginseng) 10 g, Pueraria (Gegen, Radix Puerariae) 25 g, *Salvia *Root (Danshen, Radix Salviae Miltiorrhizae) 15 g, Cassia Twig (Guizhi, Ramulus Cinnamomi) 10 g, Chinese Thorowax Root (Chaihu, Radix Bupleuri) 10 g, Turmeric Tuber (Yujin, Tuber Curcumae) 10 g, *Corydalis* Rhizome (Yanhusuo, *Corydalis* Rhizome) 10 g, White Peony Root (Baishao, Radix Albus Paeoniae Lactiflorae) 20 g

Shi et al., 2013 [25]	Modified GLXBBX decoction	Medicinal Changium Root (Shenqu, Medicated Leaven) 15 g, *Codonopsis* Root (Dangshen, Radix Codonopsitis Pilosulae) 15 g, Root of Membranous Milk Vetch (Huanqi, *Astragalus membranaceus*) 15 g, *Coix* Seed (Yiyiren, Semen Coicis) 20 g, *Salvia *Root (Danshen, Radix Salviae Miltiorrhizae) 30 g, Snake Gourd Fruit (Gualou, *Trichosanthes kirilowii *Maxim.) 12 g, Safflower (Honghua, *Carthamus tinctorius*) 12 g, *Pinellia ternata* (Banxia, Rhizoma Pinelliae) 12 g, *Allium macrostemon* Bunge (Xiebai, *Allium macrostemon*) 12 g, Immature Bitter Orange (Zhishi, Fructus Aurantii Immaturus) 10 g

Si and Yin, 2012 [26]	Modified GLXBBX decoction	Snake Gourd Fruit (Gualou, *Trichosanthes kirilowii *Maxim.) 30 g, *Allium macrostemon* Bunge (Xiebai, *Allium macrostemon*) 15 g, *Pinellia ternata* (Banxia, Rhizoma Pinelliae) 12 g, Tangerine Peel (Chenpi, Pericarpium Citri Reticulatae) 12 g, Sclerotium of Tuckahoe (Fuling, Scierotium Poriae Cocos) 15 g, *Amomum cardamomum* (Doukou, Amomum Kravanh) 12 g, Cassia Twig (Guizhi, Ramulus Cinnamomi) 6 g, *Salvia *Root (Danshen, Radix Salviae Miltiorrhizae) 20 g, Szechuan Lovage Root (Chuanxiong, Rhizoma Ligustici Chuanxiong) 15 g, Root of Common Peony (Chishao, Radix Paeoniae Rubra) 15 g, Chinese Angelica Root (Danggui, Radix Angelicae Sinensis) 15 g, *Crataegus* Fruit (Shanzha, Crataegi Fructus) 10 g, Medicinal Changium Root (Shenqu, Medicated Leaven) 10 g, Fructus Hordei Germinatus (Maiya, malt) 10 g

Wang, 2012 [27]	Modified GLXBBX decoction	Snake Gourd Fruit (Gualou, *Trichosanthes kirilowii *Maxim.) 20 g, *Allium macrostemon* Bunge (Xiebai, *Allium macrostemon*) 10 g, *Pinellia ternata* (Banxia, Rhizoma Pinelliae) 10 g, Dried Ginger (Ganjiang, Rhizoma Zingiberis) 10 g, Tangerine Peel (Chenpi, Pericarpium Citri Reticulatae) 10 g, *Amomum cardamomum* (Doukou, Amomum Kravanh) 6 g, *Acorus gramineus* (Shichangpu, Rhizoma Acori Tatarinowii) 10 g, Immature Bitter Orange (Zhishi, Fructus Aurantii Immaturus) 10 g

Zhang and Zhu, 2003 [28]	Modified GLXBBX decoction	Snake Gourd Fruit (Gualou, *Trichosanthes kirilowii *Maxim.) 30 g, *Allium macrostemon* Bunge (Xiebai, *Allium macrostemon*) 15 g, *Pinellia ternata* (Banxia, Rhizoma Pinelliae) 15 g, *Salvia *Root (Danshen, Radix Salviae Miltiorrhizae) 15 g, Root of Common Peony (Chishao, Radix Paeoniae Rubra) 12 g, Root of Membranous Milk Vetch (Huanqi, *Astragalus membranaceus*) 20 g, *Codonopsis* Root (Dangshen, Radix Codonopsitis Pilosulae) 20 g

Fang, 2011 [29]	Modified GLXBBX decoction	Snake Gourd Fruit (Gualou, *Trichosanthes kirilowii *Maxim.) 30 g, Bamboo Shavings (Zhuru, Bambusae Caulis in Taeniam) 12 g, Tangerine Peel (Chenpi, Pericarpium Citri Reticulatae) 9 g, Pueraria (Gegen, Radix Puerariae) 25 g, *Salvia *Root (Danshen, Radix Salviae Miltiorrhizae) 15 g, Sclerotium of Tuckahoe (Fuling, Scierotium Poriae Cocos) 15 g, *Allium macrostemon* Bunge (Xiebai, *Allium macrostemon*) 15 g, *Codonopsis* Root (Dangshen, Radix Codonopsitis Pilosulae) 10 g, *Pinellia ternata* (Banxia, Rhizoma Pinelliae) 10 g, Cassia Twig (Guizhi, Ramulus Cinnamomi) 10 g, Chinese Thorowax Root (Chaihu, Radix Bupleuri) 10 g, Turmeric Tuber (Yujin, Tuber Curcumae) 10 g, *Corydalis* Rhizome (Yanhusuo, *Corydalis* Rhizome) 10 g, White Peony Root (Baishao, Radix Albus Paeoniae Lactiflorae) 20 g

Zhu, 2012 [30]	Modified GLXBBX decoction	*Codonopsis* Root (Dangshen, Radix Codonopsitis Pilosulae) 10 g, Pueraria (Gegen, Radix Puerariae) 25 g, *Pinellia ternata* (Banxia, Rhizoma Pinelliae) 10 g, *Salvia *Root (Danshen, Radix Salviae Miltiorrhizae) 15 g, Cassia Twig (Guizhi, Ramulus Cinnamomi) 10 g, Bamboo Shavings (Zhuru, Bambusae Caulis in Taeniam) 12 g, Snake Gourd Fruit (Gualou, *Trichosanthes kirilowii *Maxim.) 30 g, White Peony Root (Baishao, Radix Albus Paeoniae Lactiflorae) 20 g, Turmeric Tuber (Yujin, Tuber Curcumae) 10 g, *Allium macrostemon* Bunge (Xiebai, *Allium macrostemon*) 15 g, Chinese Thorowax Root (Chaihu, Radix Bupleuri) 10 g, Tangerine Peel (Chenpi, Pericarpium Citri Reticulatae) 9 g, Sclerotium of Tuckahoe (Fuling, Scierotium Poriae Cocos) 15 g, *Corydalis* Rhizome (Yanhusuo, *Corydalis* Rhizome) 10 g

Li and Cai, 2011 [31]	Modified GLXBBX decoction	Snake Gourd Fruit (Gualou, *Trichosanthes kirilowii *Maxim.) 15 g, *Allium macrostemon* Bunge (Xiebai, *Allium macrostemon*) 8 g, Immature Bitter Orange (Zhishi, Fructus Aurantii Immaturus) 9 g, Cassia Twig (Guizhi, Ramulus Cinnamomi) 9 g, *Pinellia ternata* (Banxia, Rhizoma Pinelliae) 9 g, Root of Balloonflower (Jiegeng, Platycodon grandiflorum) 4.5 g, processed aconite (Fuzi, Radix Lateralis Praeparatus Aconiti Carmichaeli) 1.5~30 g, *Salvia *Root (Danshen, Radix Salviae Miltiorrhizae) 30 g

Zhang, 2015 [32]	Modified GLXBBX decoction	Snake Gourd Fruit (Gualou, *Trichosanthes kirilowii *Maxim.) 20 g, *Allium macrostemon* Bunge (Xiebai, *Allium macrostemon*) 10 g, *Pinellia ternata* (Banxia, Rhizoma Pinelliae) 10 g, *Salvia *Root (Danshen, Radix Salviae Miltiorrhizae) 15 g, Root of Common Peony (Chishao, Radix Paeoniae Rubra) 10 g, Notoginseng Root (Sanqifen, *Panax notoginseng* powder) 3 g, Sclerotium of Tuckahoe (Fuling, Scierotium Poriae Cocos) 15 g, Turmeric Tuber (Yujin, Tuber Curcumae) 10 g, White Sandalwood (Tanxiang, Sandalwood) 10 g, *Acorus gramineus* (Shichangpu, Rhizoma Acori Tatarinowii) 15 g, Szechuan Lovage Root (Chuanxiong, Rhizoma Ligustici Chuanxiong) 10 g, Pueraria (Gegen, Radix Puerariae) 15 g

Ma, 2015 [33]	Modified GLXBBX decoction	Snake Gourd Fruit (Gualou, *Trichosanthes kirilowii *Maxim.) 30 g, Pueraria (Gegen, Radix Puerariae) 25 g, White Peony Root (Baishao, Radix Albus Paeoniae Lactiflorae) 20 g, Bamboo Shavings (Zhuru, Bambusae Caulis in Taeniam) 12 g, Tangerine Peel (Chenpi, Pericarpium Citri Reticulatae) 9 g, Sclerotium of Tuckahoe (Fuling, Scierotium Poriae Cocos) 15 g, *Allium macrostemon* Bunge (Xiebai, *Allium macrostemon*) 15 g, *Salvia *Root (Danshen, Radix Salviae Miltiorrhizae) 15 g, Chinese Thorowax Root (Chaihu, Radix Bupleuri) 10 g, *Codonopsis* Root (Dangshen, Radix Codonopsitis Pilosulae) 10 g, Cassia Twig (Guizhi, Ramulus Cinnamomi) 10 g, *Pinellia ternata* (Banxia, Rhizoma Pinelliae) 10 g, *Corydalis* Rhizome (Yanhusuo, *Corydalis* Rhizome) 10 g, Turmeric Tuber (Yujin, Tuber Curcumae) 10 g

Yang et al., 2015 [34]	Modified GLXBBX decoction	Snake Gourd Fruit (Gualou, *Trichosanthes kirilowii *Maxim.) 25 g, *Allium macrostemon* Bunge (Xiebai, *Allium macrostemon*) 15 g, *Pinellia ternata* (Banxia, Rhizoma Pinelliae) 10 g, *Codonopsis* Root (Dangshen, Radix Codonopsitis Pilosulae) 10 g, Cassia Twig (Guizhi, Ramulus Cinnamomi) 10 g, Turmeric Tuber (Yujin, Tuber Curcumae) 10 g, Peach Seed (Taoren, Peach Keruel) 10 g, Safflower (Honghua, *Carthamus tinctorius*) 20 g, *Corydalis* Rhizome (Yanhusuo, *Corydalis* Rhizome) 10 g, Root of Membranous Milk Vetch (Huanqi, *Astragalus membranaceus*) 20 g

Tong and Xiong, 2013 [35]	Modified GLXBBX decoction	Snake Gourd Fruit (Gualou, *Trichosanthes kirilowii *Maxim.) 12 g, Bamboo Shavings (Zhuru, Bambusae Caulis in Taeniam) 12 g, Sclerotium of Tuckahoe (Fuling, Scierotium Poriae Cocos) 12 g, *Allium macrostemon* Bunge (Xiebai, *Allium macrostemon*) 9 g, *Pinellia ternata* (Banxia, Rhizoma Pinelliae) 9 g, Turmeric Tuber (Yujin, Tuber Curcumae) 9 g, *Acorus gramineus* (Shichangpu, Rhizoma Acori Tatarinowii) 9 g, *Salvia *Root (Danshen, Radix Salviae Miltiorrhizae) 15 g, Tangerine Peel (Chenpi, Pericarpium Citri Reticulatae) 6 g, Wrinkled Giant Hyssop (Huoxiang, Agastache rugosus) 6 g, Szechuan Lovage Root (Chuanxiong, Rhizoma Ligustici Chuanxiong) 6 g

Wang, 2015 [36]	Modified GLXBBX decoction	Snake Gourd Fruit (Gualou, *Trichosanthes kirilowii *Maxim.) 20 g, *Allium macrostemon* Bunge (Xiebai, *Allium macrostemon*) 20 g, *Pinellia ternata* (Banxia, Rhizoma Pinelliae) 10 g, Cassia Twig (Guizhi, Ramulus Cinnamomi) 10 g, Sclerotium of Tuckahoe (Fuling, Scierotium Poriae Cocos) 20 g, Root of Common Peony (Chishao, Radix Paeoniae Rubra) 10 g, Pueraria (Gegen, Radix Puerariae) 20 g, Peach Seed (Taoren, Peach Keruel) 15 g, Chinese Angelica Root (Danggui, Radix Angelicae Sinensis) 20 g, Szechuan Lovage Root (Chuanxiong, Rhizoma Ligustici Chuanxiong) 30 g, Immature Bitter Orange (Zhishi, Fructus Aurantii Immaturus) 10 g, Safflower (Honghua, *Carthamus tinctorius*) 20 g, Turmeric Tuber (Yujin, Tuber Curcumae) 20 g, Scorpion (Quanxie, *Buthus martensii* Karsch) 10 g, *Salvia *Root (Danshen, Radix Salviae Miltiorrhizae) 30 g, Liquorice Root (Gancao, Radix Glycyrrhizae) 20 g

Yang, 2015 [37]	Modified GLXBBX decoction	Snake Gourd Fruit (Gualou, *Trichosanthes kirilowii *Maxim.) 30 g, *Allium macrostemon* Bunge (Xiebai, *Allium macrostemon*) 15 g, *Pinellia ternata* (Banxia, Rhizoma Pinelliae) 10 g, Pueraria (Gegen, Radix Puerariae) 25 g, White Peony Root (Baishao, Radix Albus Paeoniae Lactiflorae) 20 g, *Salvia *Root (Danshen, Radix Salviae Miltiorrhizae) 15 g, Sclerotium of Tuckahoe (Fuling, Scierotium Poriae Cocos) 15 g, Bamboo Shavings (Zhuru, Bambusae Caulis in Taeniam) 12 g, *Codonopsis* Root (Dangshen, Radix Codonopsitis Pilosulae) 10 g, Cassia Twig (Guizhi, Ramulus Cinnamomi) 10 g, Turmeric Tuber (Yujin, Tuber Curcumae) 10 g, Chinese Thorowax Root (Chaihu, Radix Bupleuri) 10 g, *Corydalis* Rhizome (Yanhusuo, *Corydalis* Rhizome) 10 g, Tangerine Peel (Chenpi, Pericarpium Citri Reticulatae) 9 g

**Table 3:** The effect of GLXBBX for SAP or UAP group, outcome = RAS.

Study ID	Response rate% (response/*N*)		Therapeutic gain, %	NNT	RR
	Experimental	Control			
Shi et al., 2013 [25]	93.33 (42/45)	75.56 (34/45)	17.77	5.63	1.24
Wang, 2012 [27]	94.12 (64/68)	85.71 (36/42)	8.41	11.89	1.10
Zhu, 2012 [30]	88.00 (44/50)	66.67 (20/30)	21.33	4.69	1.32
Li and Cai, 2011 [31]	95.24 (40/42)	80.00 (32/40)	15.24	6.56	1.19
Zhang, 2015 [32]	90.48 (38/42)	71.43 (30/42)	19.05	5.25	1.27
Ma, 2015 [33]	92.73 (51/55)	76.36 (42/55)	16.37	6.11	1.21
Yang et al., 2015 [34]	90.48 (38/42)	80.95 (34/42)	9.53	10.49	1.12
Yang, 2015 [37]	90.00 (36/40)	72.50 (29/40)	17.5	5.71	1.24
Pooled RR	91.93 (353/384)	76.49 (257/336)	15.44	6.48	1.20

**Table 4:** The effect of GLXBBX for SAP or UAP group, outcome = ECG.

Study ID	Response rate% (response/*N*)		Therapeutic gain, %	NNT	RR
	Experimental	Control			
Shi et al., 2013 [25]	88.89 (40/45)	71.11 (32/45)	17.78	5.62	1.25
Zhang, 2015 [32]	73.81 (31/42)	54.76 (23/42)	19.05	5.25	1.35
Ma, 2015 [33]	96.36 (53/55)	80.00 (44/55)	16.36	6.11	1.20
Yang et al., 2015 [34]	88.10 (37/42)	76.19 (32/42)	11.90	8.40	1.16
Pooled RR	87.5 (161/184)	71.20 (131/184)	16.30	6.13	1.23

**Table 5:** The effect of GLXBBX for SAP, outcome = RAS.

Study ID	Response rate% (response/*N*)		Therapeutic gain, %	NNT	RR
	Experimental	Control			
Zhang and Zhu, 2003 [28]	96.67 (58/60)	86.67 (52/60)	10.00	10.00	1.12
Fang, 2011 [29]	79.41 (27/34)	66.67 (20/30)	12.74	7.85	1.19
Wang, 2015 [36]	98.10 (103/105)	88.57 (93/105)	9.53	10.49	1.11
Pooled RR	94.47 (188/199)	84.62 (165/195)	9.85	10.15	1.12

**Table 6:** The effect of GLXBBX for SAP, outcome = ECG.

Study ID	Response rate% (response/*N*)		Therapeutic gain, %	NNT	RR
	Experimental	Control			
Zhang and Zhu, 2003 [28]	71.67 (43/60)	50.00 (30/60)	21.67	4.61	1.43
Wang, 2015 [36]	97.14 (102/105)	82.86 (87/105)	14.28	7.01	1.17
Pooled RR	87.88 (145/165)	70.91 (117/165)	16.98	5.89	1.24

**Table 7:** The effect of GLXBBX for UAP, outcome = RAS.

Study ID	Response rate% (response/*N*)		Therapeutic gain, %	NNT	RR
	Experimental	Control			
Si and Yin, 2012 [26]	93.33 (28/30)	76.67 (23/30)	16.66	6.00	1.22
Tong and Xiong, 2013 [35]	93.75 (30/32)	87.10 (27/31)	6.65	15.04	1.08
Pooled RR	93.55 (58/62)	81.97 (50/61)	11.59	8.63	1.14

**Table 8:** The effect of GLXBBX for UAP, outcome = ECG.

Study ID	Response rate% (response/*N*)		Therapeutic gain, %	NNT	RR
	Experimental	Control			
Si and Yin, 2012 [26]	80.00 (24/30)	63.33 (19/30)	16.67	6.00	1.26
Pooled RR	80.00 (24/30)	63.33 (19/30)	16.67	6.00	1.26
